# PPP1R12A Mutation Presenting With Congenital Jejunal Atresia and Short Stature: A Pediatric Endocrinology Case Report

**DOI:** 10.1155/crpe/2247764

**Published:** 2026-01-28

**Authors:** Rosita Saul, Maya David, Jordin Frasch, Pedro A. Sanchez-Lara, Bahareh M. Schweiger

**Affiliations:** ^1^ Department of Osteopathic Medical Education, Dr. Kiran C. Patel College of Osteopathic Medicine, Nova Southeastern University, Fort Lauderdale, Florida, USA, nova.edu; ^2^ Division of Pediatric Endocrinology, Guerin Children’s, Cedars-Sinai Medical Center, Los Angeles, California, USA, cedars-sinai.edu; ^3^ Division of Pediatric Genetics, Guerin Children’s, Cedars-Sinai Medical Center, Los Angeles, California, USA, cedars-sinai.edu

## Abstract

We report an 11‐year‐old Hispanic male with a PPP1R12A gene de novo heterozygous likely pathogenic mutation, p. (Gln13Arg) (CAG>CGG), c.38 *A* > *G* in Exon 1 (NM_002480.2), detected on whole‐exome trio sequencing during his short‐stature evaluation. His medical history is remarkable for congenital jejunal atresia diagnosed prenatally and repaired surgically shortly after birth. Notably, he lacks genitourinary anomalies, which are frequently described in individuals with PPP1R12A‐related urogenital and brain malformation syndrome (UBMS). An endocrine evaluation revealed growth hormone deficiency with an ectopic posterior pituitary gland and an interrupted pituitary stalk. Despite these findings, his neurodevelopment is advanced compared to peers without any concern for intellectual disability. His most recent gastrointestinal and nutritional workup was normal, and he is demonstrating excellent linear growth and response to somatropin therapy. This case broadens the phenotypic spectrum associated with PPP1R12A mutations by highlighting isolated growth hormone deficiency and jejunal atresia in the absence of genitourinary and neurodevelopmental anomalies. We emphasize the importance of multidisciplinary monitoring and an early endocrine referral in patients with PPP1R12A variants presenting with short stature.

## 1. Introduction

PPP1R12A encodes the myosin phosphatase target Subunit 1 (*MYPT1*), a regulatory component of myosin phosphatase that modulates actin–myosin interactions essential for cell shape, motility, and contractility [1]. Pathogenic variants in PPP1R12A have been associated with an autosomal dominant neurodevelopmental disorder characterized by variable combinations of genitourinary (GU) anomalies, midline and cortical brain malformations, and intellectual disability [2, 3]. Reported clinical features across affected individuals are summarized in Table 1, and the phenotypic spectrum continues to expand as additional clinical reports emerge.

**Table 1:** Clinical features reported in individuals with PPP1R12A variants [3]. Summary of phenotypic findings, including central nervous system, urogenital, gastrointestinal, and limb anomalies, along with associated genetic findings and expression profile.

Clinical feature	Description/examples	Frequency/notes
Midline brain malformations	Holoprosencephaly, other forebrain division defects	∼5/12 cases
Urogenital anomalies	Disorders of sex development, ambiguous genitalia, hypospadias, lower urinary tract malformations	∼9/12 cases
Combined brain and urogenital	Both CNS and urogenital anomalies in same individual	2/12 cases
Gastrointestinal anomalies	Omphalocele, jejunal/ileal atresia, aberrant mesenteric blood supply	Variable
Limb anomalies	Syndactyly	Occasional
Genetic findings	De novo loss‐of‐function variants (stop gain, frameshift, splice site)	All reported cases
Expression profile	PPP1R12A expressed in prosencephalic neural folds and lower urinary tract during embryogenesis	Pathogenic mechanism

Gastrointestinal malformations, though less common, have been increasingly recognized. Recent case reports describe intestinal atresia in association with PPP1R12A, the c.3092 *A* > *T* variant, including a patient with Type IIIb jejunal atresia, imperforate anus, and vaginal atresia, and another with ileal and esophageal atresia [4, 5]. Together, these findings suggest a broader role for PPP1R12A in gastrointestinal and reproductive tract development than previously appreciated.

Endocrine involvement is rare but noteworthy. Growth hormone deficiency (GHD) and pituitary malformations have been reported in a small subset of patients, usually in association with global delays or broader syndromic features [3]. The relationship between PPP1R12A and pituitary development remains incompletely understood. A recent study examined patients with pituitary stalk interruption syndrome (PSIS)—a phenotype that can overlap with midline anomalies seen in PPP1R12A‐related disorder. Although no pathogenic PPP1R12A variants were identified in their cohort, the study highlights both the genetic heterogeneity underlying midline brain malformations and the importance of considering PPP1R12A in the differential diagnosis [6].

Recent reports have continued to expand the clinical and mutational spectrum of PPP1R12A‐related disorder. Variants can impact sex development, as illustrated by a patient with a de novo frameshift mutation who presented with sex reversal, testicular atrophy, hypertelorism, and multiple skin hemangiomas [7]. Pathogenic variants may also involve noncoding regions, as demonstrated by a neonate with congenital micropenis due to a de novo splice variant (c.2666+3*A* > *G*); functional assays confirmed aberrant splicing and exon skipping [8]. Neurologic manifestations can arise as well, as evidenced by a neonate with infantile epilepsy and urogenital/brain malformations carrying a novel nonsense variant (c.2533*C* > *T*, p.Arg845Ter), with functional studies showing a truncated protein and impaired interaction with PPP1CB [9].

Here, we report a patient with a heterozygous pathogenic PPP1R12A variant presenting with congenital jejunal atresia and GHD due to ectopic posterior pituitary in the absence of GU anomalies or neurodevelopmental impairment. This case adds to the emerging literature by illustrating an atypical presentation and further broadening the clinical spectrum associated with PPP1R12A‐related diseases.

## 2. Case Presentation

The patient is an 11‐year‐old Hispanic male born at 33 weeks and 2 days of gestation via repeat low transverse cesarean section to a 38‐year‐old mother after a pregnancy complicated by preterm premature rupture of membranes. Prenatal ultrasound at 24 weeks 4 days revealed dilated bowel loops suggestive of jejunal atresia or obstruction, with progressive polyhydramnios noted at 28 weeks. His birth weight was 2.401 kg (10th percentile), and length was 48 cm (25th percentile). Postnatally, he underwent resection of the atretic jejunal segment and reanastomosis on Day 2 of life, followed by a 45‐day NICU stay during which he received total parenteral nutrition with slow advancement of feedings to breast milk. An echocardiogram identified mild peripheral left pulmonary artery stenosis, a patent foramen ovale, and abdominal aortic flow reversal. He also had anemia in the newborn period that resolved without intervention.

Developmentally, the patient had early torticollis requiring physical therapy and experienced mild motor delays, including low abdominal tone and delayed crawling. He experienced fine motor delays throughout preschool and kindergarten, but these improved with intervention. Academically, he performed above average in math and reading skills but was held back a year primarily for short‐stature–related bullying.

Growth data over several years revealed persistent short stature. Family history was negative for short stature, congenital anomalies, developmental delay, or endocrine disorders. His past medical history was otherwise unremarkable. His mid‐parental height was calculated at 179.2 cm (70.6 inches). Height percentiles ranged from the 0.37th to the 3.57th percentile between the ages of 8 and 11 years. Growth velocity fluctuated from approximately 6–7.8 cm/year.

Endocrine evaluation was initiated due to the patient’s height tracking near the third percentile from ages 3–7 years, followed by a decline to the 0.37th percentile by 8 years 11 months. Workup revealed a bone age of 7–8 years at chronological age 8 years 4 months, revealing a skeletal age of 7‐8 years. Laboratory testing showed normal thyroid function, a negative celiac screen, normal inflammatory markers, low serum iron, and mildly decreased IGF‐1 (88 ng/mL). Growth hormone stimulation testing demonstrated peak growth hormone levels of 6.9 ng/mL with clonidine and 1.7 ng/mL with glucagon, consistent with GHD. Brain MRI revealed a small anterior pituitary, an interrupted infundibulum, and an ectopic posterior pituitary.

**Figure 1:**
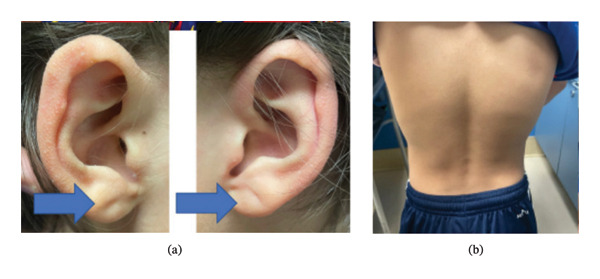
Clinical photographs obtained during genetics evaluation demonstrating subtle dysmorphic features. (a) Bilateral earlobe creases and pinna abnormalities. (b) Minor thoracic asymmetry.

Due to short stature with iron deficiency and minor dysmorphisms, the patient was referred to genetics for evaluation. On physical examination, the geneticist noted subtle dysmorphic features, including bilateral earlobe creases, slight pinna abnormalities, and minor thoracic asymmetry, as shown in Figure 1. Genetic testing by sequencing identified a PPP1R12A gene de novo heterozygous likely pathogenic mutation, p. (Gln13Arg) (CAG > CGG): c.38 *A* > *G* in Exon 1), which encodes a subunit of myosin phosphatase that regulates actin–myosin interactions downstream of the rho GTPase pathway. Variants in this gene have been associated with a spectrum of GU and brain malformations. Notably, this patient exhibits no GU abnormalities. His pubertal examination revealed Tanner Stage I pubic hair, bilateral testicular volumes of 2 mL, normal genitalia without gynecomastia, and a stretched penile length of 4 cm.

He is followed by pediatric gastroenterology, with a normal workup at age 8, including stool studies and an upper GI series with small bowel follow‐through. He currently remains free of gastrointestinal symptoms related to his history of jejunal atresia and reanastomosis. Although no specific long‐term surveillance for surgical complications (such as adhesions or strictures) has been recommended or documented, he continues to be seen by GI for management of functional constipation consistent with IBS‐constipation, with no additional concerns raised regarding his prior jejunal atresia.

**Figure 2:**
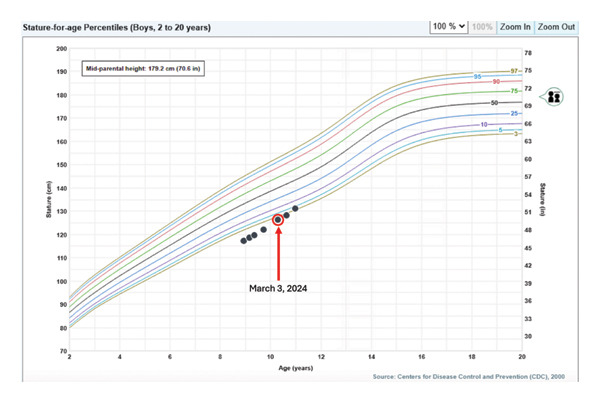
Stature‐for‐age chart demonstrating the patient’s height at each clinic visit: 117.3 cm (0.37th percentile, January 2023), 118.6 cm (April 2023), 119.7 cm (June 2023), 122.1 cm (November 2023), 126.3 cm (May 2024), 128.3 cm (September 2024), and 131 cm (3.57th percentile, January 2025). Growth hormone therapy with somatotropin was initiated on March 3, 2024, after which an upward shift in growth trajectory was observed (as indicated by the red circle).

**Figure 3:**
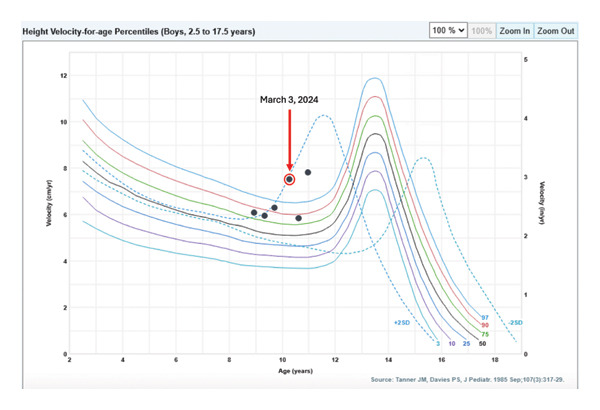
Height velocity chart demonstrating the patient’s annualized growth rates at each clinic visit: 6.075 cm/yr (January 2023), 5.963 cm/yr (June 2023), 6.306 cm/yr (November 2023), 7.52 cm/yr (May 2024), 5.844 cm/yr (September 2024), and 7.827 cm/yr (January 2025). Growth hormone therapy with somatotropin was initiated on March 3, 2024, after which an overall increase in velocity was observed (as indicated by the red circle).

Growth hormone therapy was initiated at 0.8 mg daily (0.25 mg/kg/week), consistent with Pediatric Endocrine Society–recommended weight‐based dosing ranges and at the upper end of standard prepubertal dosing; IGF‐1 levels remained within the normal range, and the patient demonstrated an excellent growth response [10]. Pubertal onset and progression have been age‐appropriate to date. Height‐for‐age and velocity charts are shown in Figures 2 and 3, demonstrating increased growth following initiation of growth hormone therapy.

## 3. Discussion

This patient presents a novel clinical profile of PPP1R12A‐related disorder, expanding the phenotypic spectrum by highlighting isolated GHD and jejunal atresia without GU or neurodevelopmental abnormalities. Published cohorts predominantly report GU malformations, brain structural anomalies including holoprosencephaly, and neurodevelopmental delay [2, 3]. Our patient’s absence of GU defects and advanced neurodevelopment contrasts with the canonical UBMS phenotype.

The ectopic posterior pituitary and interrupted pituitary stalk on MRI are hallmarks of congenital GHD and hypopituitarism [11–13], features not previously emphasized in PPP1R12A mutation cases. Given the role of PPP1R12A in regulating smooth muscle contractility and cellular architecture, the association with jejunal atresia is biologically plausible and matches prior reports of gastrointestinal malformations [2].

This case underscores the need for multidisciplinary care and vigilance for endocrine dysfunction in patients with PPP1R12A mutations, especially when unexplained short stature or pituitary anomalies are present. The absence of GU anomalies does not exclude the diagnosis, and longitudinal surveillance for pubertal development and gonadal function remains prudent.

## 4. Conclusion

Our report adds to growing evidence that PPP1R12A‐related disorders exhibit wide phenotypic variability. We document a case of congenital jejunal atresia and isolated GHD with pituitary abnormalities in a child lacking the GU and neurodevelopmental defects typically associated with this syndrome. Early endocrine referral and genetic evaluation are vital in patients presenting with unexplained growth failure and congenital anomalies. Continued follow‐up will clarify long‐term endocrine and developmental outcomes.

## Funding

No funding was received for this manuscript.

## Consent

Verbal consent has been obtained from the patients as there are no patient identifiable data included in this case report/series.

## Conflicts of Interest

The authors declare no conflicts of interest.

## Data Availability

Data sharing is not applicable to this article as no datasets were generated or analyzed during the current study.
